# The Diagnosis and Management of Herpes Simplex Pneumonia in the Critical Care Setting: A Comprehensive Review

**DOI:** 10.7759/cureus.43224

**Published:** 2023-08-09

**Authors:** Ramakanth Pata, Praveen Datar

**Affiliations:** 1 Pulmonary and Critical Care Medicine, One Brooklyn Health, New York, USA; 2 Pulmonary and Critical Care Medicine, University of Cincinnati Medical Center, Cincinatti, USA; 3 Pulmonary and Critical Care Medicine, Ozarks Medical Center, West Plains, USA

**Keywords:** acute respiratory distress syndrome, ventilator-associated pneumonia, disseminated herpes, prognosis, treatment, diagnosis, immunocompromised, hsv pneumonitis, hsv pneumonia, herpes simplex virus

## Abstract

Herpes simplex virus (HSV) belongs to the *Herpesviridae* family and is divided into two subtypes: HSV-1 and HSV-2. It is known that herpesviruses lie dormant in neural ganglion cells and are reactivated during times of stress, trauma, fever, and immunosuppression. While HSV primarily causes mucosal infections such as cold sores or upper respiratory tract manifestations, it can also lead to serious, life-threatening infections, particularly in immunocompromised patients. Although HSV is occasionally detected in airway samples from critically ill patients, true HSV pneumonia is rare. HSV pneumonia is thought to result from the aspiration of salivary secretions that can travel from the pharynx and tracheobronchial areas to the lungs. It can be difficult to diagnose, and the presence of HSV in respiratory specimens does not necessarily indicate true infection. Treatment with antiviral drugs such as acyclovir should be considered based on the clinical presentation, corroborative findings, and the presence of cytopathological changes in the bronchoalveolar specimen. The prognosis of HSV pneumonia is generally poor and early detection is critical for better outcomes. This review discusses the risk factors, clinical presentation, diagnosis, treatment, and prognosis of HSV pneumonia and emphasizes the importance of distinguishing between true infection and carrier status.

## Introduction and background

Belonging to the *Herpesviridae* family, the herpes simplex virus (HSV) is classified into two subtypes: HSV-1 and HSV-2. HSV is known to primarily cause mucosal infections, which have been described since ancient times. HSV remains dormant in the neural ganglion cells, with reactivation occurring during stress, trauma, fever, and periods of immunosuppression. Most commonly, HSV-1 presents as "cold sores", herpes labialis, or upper airway manifestations such as gingivostomatitis and pharyngitis. HSV can also lead to severe, life-threatening infections, especially in immunocompromised patients [1].

Although frequently isolated in broncho-alveolar lavage of critically ill patients, especially with prolonged intubations, true HSV pneumonia is extremely rare. When detected in respiratory specimens of critically ill immunocompetent patients, it is generally considered an innocent bystander. However, a true infection such as HSV pneumonia or viral pneumonitis (referred to as pneumonitis if inflammatory sequelae predominate) can occur in immunosuppressed individuals. In such cases, mortality tends to be very high [2].

## Review

Epidemiology

HSV-1 may be isolated in 5-64% of intensive care unit (ICU) patients from the upper respiratory tract. One observational study revealed that the prevalence of HSV detection increases as the duration of mechanical ventilation increases, with 54% from upper respiratory samples and 64% from lower respiratory samples in patients who have been intubated for more than five days [3-5]. Whether positive HSV in these samples represents reactivation or a new infection is currently uncertain.

Pathogenesis

The pathogenesis of HSV pneumonia is thought to be due to the aspiration of salivary secretions, which has the potential to involve pharyngeal and tracheobronchial regions and ultimately progress to the lungs, causing pneumonia [3]. Occasionally, neurogenic or hematogenous spread has been described with disseminated herpes infection [4]. However, local pulmonary reactivation and primary hematogenous dissemination cannot be totally excluded.

Risk factors

Suppression of cell-mediated immunity is the primary risk factor for reactivation of HSV. The factors that may suppress the immune system include chemotherapy, burns, and trauma [6]. Based on the level of immunocompetence, reactivation of HSV can present with a spectrum of severity, from an innocuous positivity in diagnostic samples and mucositis to life-threatening disseminated infections. Frequently, most individuals with anticipated prolonged immunosuppression, such as transplant recipients, therefore receive prophylaxis with acyclovir.

In critically ill patients, older age, smoking history, and prolonged intubation are risk factors for mortality related to herpes pneumonia, even in the immunocompetent group [7]. Mechanical ventilation for more than five days is not only associated with the presence of HSV in lower respiratory samples but has also been demonstrated as a risk factor for HSV bronchopneumonia with worse outcomes. HSV bronchopneumonia should always be considered one of the causes of ventilator-associated pneumonia in patients who deteriorate despite adequate antibiotic therapy [4,5].

Clinical presentation

HSV pneumonia typically presents with prodromal symptoms such as fever, myalgia, and rapidly worsening respiratory failure. While the presence of mucocutaneous lesions may not always be evident, their absence does not exclude the possibility of HSV infection [2,8]. Patients may initially present with other symptoms, such as neutropenic fever, abdominal pain, and diarrhea, leading to a delay in diagnosing HSV pneumonia. Therefore, a high index of suspicion is crucial for timely recognition and appropriate management, especially if unresponsive to broad-spectrum antibiotic therapy for presumed neutropenic enterocolitis. In those cases, a computed tomography (CT) scan of the chest, abdomen, and pelvis will guide further diagnostic measures such as bronchoscopy.

Diagnosis

Diagnosing HSV pneumonia in critically ill patients can be challenging. The detection of HSV in respiratory samples does not necessarily indicate a true infection, as HSV can exist as a carrier state ("the innocent bystander"). HSV may be isolated in respiratory secretions in about 25-50% of patients, especially with prolonged ventilation [2,4]. Various respiratory specimens can be obtained, and it is important to note that traditional respiratory viral panels (such as nasal or nasopharyngeal swabs) do not detect HSV; hence, a separate and specific test should be ordered to identify HSV. Sputum samples cannot differentiate between upper or lower respiratory tract origin [3]. Although the true lower respiratory tract origin may be confirmed using broncho-alveolar lavage (BAL), bronchial washings tend to have a higher yield than BAL for HSV infection [5]. Serology cannot differentiate between primary, past, or recurrent infections [2]. Viremia is diagnosed with a blood sample and should always be considered in all cases of HSV pneumonia.

HSV may be detected by a variety of methods, such as the nucleic acid amplification test (NAAT), viral cultures, and antigen detection methods. Due to its 100% specificity, polymerase chain reaction (PCR) has essentially replaced viral cultures, which were traditionally considered the gold standard test [9]. A positive direct fluorescent antibody test on BAL or bronchial washings may also be utilized to diagnose lower respiratory tract involvement [8,10]. One of the most important clues to differentiate infection from a carrier state is to look for cytopathological changes seen on BAL specimens. Although lung biopsy is theoretically considered the most definitive way to establish a diagnosis of HSV pneumonia, this may not always be reliable and, in fact, may be hazardous in critically ill patients, especially those with acute respiratory distress syndrome (ARDS) while on the ventilator [11]. Once identified in a respiratory or blood sample, it may be reasonable to establish the degree of dissemination by imaging of the head (CT or MRI findings of temporal lobe involvement) and fundoscopy (e.g., acute retinal necrosis, chorioretinitis) as these findings are useful in determining the duration of therapy [9,10].

Treatment

Acyclovir administration, especially in critically ill patients with multiorgan dysfunction, can lead to adverse effects such as renal dysfunction and neurotoxicity. Hence, risk versus benefits should always be considered before deciding to treat a positive HSV sample [4].

Deciding whether to treat or not can be challenging, as HSV is known to act as an innocent bystander or carrier state. Some studies show that treatment with acyclovir does not improve outcomes such as ventilator days or mortality. Many of these studies refer to a positive sample of HSV, likely representing a carrier state rather than a true infection. Therefore, isolation of HSV in respiratory samples such as bronchial washings or BAL by itself is not necessarily an indication for treatment [4,7].

Data regarding the management of herpes pneumonia in the ICU is limited; however, it has been reported that HSV viral load decreases gradually after the initiation of acyclovir monotherapy. Studies have demonstrated that therapeutic doses of acyclovir result in lower "in hospital" and ICU mortality if given for HSV pneumonia rather than a positive sample. Therefore, clinical clues do play an important role in distinguishing a true infection from a carrier state [12].

Herpes simplex virus pneumonia should always be considered for immunocompromised hosts who have not been on acyclovir prophylaxis. In these patients, a positive sample almost always requires treatment. Acyclovir should also be considered for HSV viremia, as disseminated herpes has a high mortality rate of 30-60% [13].

In those patients whose respiratory samples, such as bronchial washings or BAL, test positive, corroborative findings such as evidence of mucosal infection like pharyngitis or tracheobronchitis, otherwise unexplained radiographic infiltrates, and continued deterioration despite adequate antibiotics for ventilator-associated pneumonia should be used to decide on the treatment. BAL should be examined for cytopathological changes that, if present, strongly support pathogenicity and warrant treatment [2,14].

One study revealed that acyclovir therapy in patients with a high viral load (> 10^5^) was associated with improvement in organ function and a longer time to death [5].

There is not enough data to suggest a confident dosing and duration of acyclovir therapy for HSV pneumonia. Due to its narrow therapeutic index, the risk of adverse effects must be balanced against high mortality due to suboptimal dosing. There is significant practice variation in terms of dosing and duration. It is recommended that the intensive care physician communicate with a critical care pharmacist and infectious disease specialist for dosing purposes. Frequently, most ICUs employ dosing similar to HSV encephalitis (10 mg/kg IV every eight hours) with varying durations ranging from 10 to 21 days. If the viral burden is low, a dose of 5 mg/kg IV every eight hours may be considered. Due to its smaller size, low protein binding capacity, and high water solubility, it is dependent on renal clearance. For the same reasons, it is eliminated in all forms of renal replacement therapy, such as intermittent hemodialysis (IHD) or continuous renal replacement therapy (CRRT). Pharmacokinetic data suggest dosing of 5-7.5 mg/kg IV every 24 hours will be sufficient in most critically ill patients on renal replacement therapy. In terms of renal elimination, a 24-hour period of CRRT is considered equivalent to a single session of IHD. If available, therapeutic drug monitoring should be considered [15].

Role of Steroids

Corticosteroids, when administered early in severe herpes-related pneumonia, may prevent the development of fibrosis. Steroids also improve outcomes in herpes-related organizing pneumonia [16]. However, due to immunosuppressive effects, steroid administration in mild disease increases severity and may result in ARDS or even dissemination causing hepatitis or encephalitis [17, 18]. Hence, the risk versus benefit must be weighed before starting steroids. The dose of corticosteroids, if considered in HSV pneumonia is not based on evidence, but based on case reports and extrapolation from other studies and depends on the clinical circumstance. There is a wide variation of practice in the dosing of steroids in severe ARDS. For severe ARDS, some intensivists use dexamethasone 20 mg/day for the first five days followed by 10 mg/day for the next five days based on the DEXA-ARDS protocol. If HSV is confirmed to be responsible for severe organizing pneumonia, then prednisone can be started at 0.5 mg/kg/day (not exceeding 60mg) with an extended taper over four to six weeks. It should be emphasized that mild to moderate cases of organizing pneumonia does not warrant steroid therapy [16].

Prophylaxis

Prophylaxis in patients at risk for herpes pneumonia, such as transplant recipients or those who are on chemotherapy or immunosuppression, decreases the incidence and associated mortality of herpes pneumonia. However, even though prolonged intubation is considered a risk factor for herpes pneumonia, routine prophylaxis is discouraged in critically ill patients unless this is continued as a part of premorbid therapy in immunosuppressed patients [2,4].

Prognosis

HSV pneumonia is associated with a poor prognosis, even in immunocompetent individuals. Prolonged intubation, chemotherapy, and neutropenia are significant risk factors for HSV pneumonia. The mortality rate among patients diagnosed with HSV pneumonia is high, with more than half succumbing to the infection. Early identification of HSV pneumonia is crucial for improving patient outcomes, including mortality and length of stay in the ICU.

HSV pneumonia is associated with a poor prognosis. Early identification is vital for patient outcomes, including mortality and length of ICU stay. In an immunocompetent patient in the ICU, a positive HSV sample is more often considered a marker than a mediator of severe illness [2].

Many studies have demonstrated that HSV bronchopneumonia has been associated with longer ventilator days, ICU length of stay, and increased mortality. Once the diagnosis of HSV pneumonia is made in critically ill patients, the level of immunocompetence does not affect hospital mortality or the number of ventilator-associated pneumonia bacterial episodes [2,4,7]. However, mechanical ventilation not only increases pneumonia by aspiration (or even microaspiration) of salivary secretions but also may increase the risk of HSV viremia, likely due to increased alveolo-capillary permeability leading to the translocation of pathogens into the systemic circulation [12].

More than half of the patients diagnosed with HSV pneumonia eventually succumb to death. The reported mortality range is up to 60-63%. Interestingly, HSV viremia was not associated with increased attributable mortality when compared to HSV pneumonia [2,12]

## Conclusions

HSV pneumonia is a severe and life-threatening condition, that primarily affects immunocompromised individuals. Prolonged intubation (> 5 days), chemotherapy, and any cause of neutropenia are strong risk factors for herpes pneumonia. A positive sample by itself does not need treatment unless it is from an immunocompromised host. Clinical correlates help in determining if a positive sample represents a carrier state or a true pathogen. Evidence of mucositis, otherwise unexplained radiographic infiltrates, and continued deterioration despite adequate antibiotics when administered for ventilator-associated pneumonia are some of the clues to consider therapy. HSV isolated in blood or BAL with evidence of cytopathological changes almost always warrants anti-viral therapy. There is some evidence that treating high viral loads may improve outcomes. Future research is needed to enhance our understanding of the optimal management strategies for HSV pneumonia.
